# Comparison of HEMOlysis Markers Among Three Pulsed Field Ablation Systems: HEMO‐PFA Study

**DOI:** 10.1111/jce.70343

**Published:** 2026-04-11

**Authors:** Kazuhisa Matsumoto, Kei Matsumoto, Naomichi Tanaka, Wataru Sasaki, Tsukasa Naganuma, Masataka Narita, Hitoshi Mori, Yoshifumi Ikeda, Takahide Arai, Shintaro Nakano, Kazuo Matsumoto, Ritsushi Kato

**Affiliations:** ^1^ Department of Cardiology Saitama Medical University, International Medical Center Saitama Japan

**Keywords:** atrial fibrillation, catheter ablation, hemolysis, myocardial injury, pulse field ablation

## Abstract

**Background:**

Pulsed field ablation (PFA) has emerged as a novel non‐thermal modality for atrial fibrillation (AF) ablation. While hemolysis is a known complication of PFA, comparative data among different systems remain limited.

**Methods:**

We prospectively enrolled 150 consecutive AF patients undergoing ablation with one of three PFA systems: FARAPULSE (FP), PulseSelect (PS), or VARIPULSE (VP) (50 cases each). Perioperative blood samples were collected pre‐procedure, immediately post‐procedure, and at 24 h. Hemolysis markers (LDH, LD2, indirect bilirubin, haptoglobin) and myocardial injury markers (CK, CK‐MB, troponin T) were measured. Procedural characteristics and complications were compared across the systems.

**Results:**

Among 144 analyzed patients, VP was associated with the shortest procedure time (76 ± 27 min vs. FP 91 ± 25 min and PS 97 ± 26 min, *p* < 0.001) and fewest applications (23 ± 5 vs. FP 40 ± 6 and PS 56 ± 26, *p* < 0.001). Hemolysis markers increased in all systems, but the magnitude varied. ΔLDH was highest with FP (95.1 ± 49.4 U/L), intermediate with PS (62.6 ± 38.1 U/L), and lowest with VP (41.3 ± 26.7 U/L; *p* < 0.001). Haptoglobin decreased most with FP (ΔHp −56.5 ± 27.2 mg/dL), followed by VP (−41.7 ± 25.8 mg/dL) and PS (−28.3 ± 21.6 mg/dL; *p* < 0.001). Myocardial injury also differed: ΔTroponin T was lowest with VP (1545 ± 920 pg/mL vs. FP 2392 ± 1123 pg/mL and PS 2128 ± 1041 pg/mL, *p* = 0.002), with a similar trend for ΔCK (141 ± 92 U/L vs. 231 ± 138 U/L and 207 ± 119 U/L, *p* < 0.001). Complications included acute kidney injury in two FP cases and cardiac tamponade in one PS case.

**Conclusions:**

Hemolysis and myocardial injury differ among PFA systems. FP was associated with the greatest changes, PS with the least hemolysis, and VP with the least myocardial injury. These differences may reflect variations in pulse output, electrode configuration, and tissue contact visualization.

## 1 Introduction

Catheter ablation has become an essential treatment for cardiac arrhythmias, with pulmonary vein isolation (PVI) being the cornerstone of atrial fibrillation (AF) ablation [1, 2, 3]. Conventionally, thermal ablation methods such as radiofrequency (RF) ablation and cryoballoon ablation have been widely employed.However, thermal ablation carries the risk of serious complications, including atrioesophageal fistula [4] and pulmonary vein stenosis [5, 6, 7] Recently, pulsed field ablation (PFA) has emerged as a novel non‐thermal modality that induces cardiomyocyte‐specific electroporation and apoptosis using high‐voltage electrical pulses of microsecond duration [8, 9]. This technique enables non‐thermal ablation, with the potential to significantly reduce the risk of atrio‐esophageal fistulae and pulmonary vein stenosis [9, 10]. Most PFA systems are approved for paroxysmal AF, and increasing evidence supports their comparable efficacy and safety to conventional thermal ablation. PFA using FARAPULSE (Boston Scientific; FP) demonstrated efficacy and safety comparable to thermal ablation, with similar arrhythmia‐free survival [11, 12, 13]. Moreover, compared with cryoballoon ablation, PFA has been associated with shorter or comparable procedure and fluoroscopy times, and a significantly lower overall complication rate [14, 15, 16]. Nevertheless, hemolysis is a recognized issue in PFA and can occasionally lead to clinically relevant acute kidney injury (AKI) [17, 18, 19, 20]. Previous studies have suggested that hemolysis is influenced by the number of applications and catheter–tissue contact [18, 21, 22], but direct comparisons of hemolysis across PFA systems remain limited [23, 24] This study was therefore designed to compare and evaluate perioperative hemolysis and myocardial injury among the three currently available PFA systems‐FP, PulseSelect (Medtronic; PS), and VARIPULSE (Johnson & Johnson; VP)‐in patients undergoing AF ablation.

## 2 Methods

### 2.1 Study Population

Between January 1 and August 1, 2025, a total of 150 consecutive patients with AF (paroxysmal and persistent) undergoing PFA at our institution were prospectively enrolled. Each of the three PFA systems—FP, PS, and VP—was used in 50 patients. Patients with persistent AF were also included in this study. When preprocedural pulmonary vein computed tomography (CT) revealed small pulmonary veins associated with a high risk of stenosis after RF ablation, operators selected PFA even in persistent AF cases.

All patients provided written informed consent to participate in this study. The study was conducted in accordance with the Declaration of Helsinki and local regulations, with approval by the institutional review board (IRB#2024‐131).

## 3 Ablation Procedure

All procedures were performed under deep sedation, using dexmedetomidine hydrochloride, propofol, and pentazocine. The bispectral index (BIS) was monitored and maintained at 40−60. Venous access was obtained via the right femoral vein, followed by transseptal puncture to enter the left atrium. Heparin was administered to maintain an activated clotting time (ACT) ≥ 350 s throughout the procedure.

In all patients, PVI was performed using PFA with a biphasic bipolar waveform, delivering microsecond‐scale pulses. Ablation with each system was performed according to the following protocol;

FARAPULSE: Four intravein applications in the basket configuration and four antral applications in the flower configuration per vein.

PulseSelect: Four intravein and four antral applications per vein.

VARIPULSE: Two intravein and two antral applications per vein.

After this standard ablation protocol, three‐dimensional mapping with a multipolar catheter was performed, and additional ablation was applied when the residual potentials were detected. When the tissue contact was deemed insufficient, additional applications were delivered at the operator's discretion. Finally, three‐dimensional mapping was repeated to confirm that complete electrical isolation of all PV had been achieved. In patients with atrial flutter identified before or during the procedure, additional linear ablation, such as cavotricuspid isthmus (CTI) ablation was performed using an irrigated‐tip RF catheter. For three‐dimensional electroanatomical mapping, the Octaray catheter (Biosense Webster, Irvine, CA, USA) was used in the VARIPULSE group, the Advisor HD Grid catheter (Abbott, Chicago, IL, USA) in the PulseSelect group, and the Orion high‐density mapping catheter (Boston Scientific, Marlborough, MA, USA) in the FARAPULSE group. For RF ablation, an irrigated DiamondTemp ablation catheter (Medtronic, Minneapolis, MN, USA) was used. Patients who underwent additional RF ablation were also included in the analysis.

## 4 Blood Sample Collection

Venous blood samples were collected at three time points: before the procedure, immediately after ablation (0 h), and on the following day (24 h). Hemolysis markers included lactate dehydrogenase (LDH), LD isoenzyme 2 (LD2), indirect bilirubin (InD‐Bil), and haptoglobin (Hp); myocardial injury markers included creatine kinase (CK), CK‐MB, and troponin T; and renal function was assessed by serum creatinine. In the present analysis, cases in which additional RF ablation was performed were also included in the assessment of hemolysis and myocardial injury markers.

## 5 Statistical Analysis

Statistical analyses were performed using SPSS version 27 software (IBM, Chicago, IL, USA). Continuous variables are expressed as mean ± SD. Intergroup differences were analyzed using one‐way analysis of variance (ANOVA) with Tukey–Kramer post hoc testing. Categorical variables were analyzed using the Chi‐square test. A *p*‐value < 0.05 was considered statistically significant.

## 6 Results

### 6.1 Patient Characteristics

Of the 150 enrolled patients, six were excluded from biomarker analysis due to blood sampling–related hemolysis (FP: 3, PS: 2, VP: 1). Thus, 144 patients were included in the analysis. Of the 144 patients, 126 underwent PVI only, while 18 received additional RF ablation. Table 1 summarizes the clinical characteristics of the entire study population and stratified by FP, PS, and VP groups. The proportion of PAF was significantly lower in the PS group (47.9%, *p* = 0.001, VP*FP: *p* = 0.001, VP*PS: *p* < 0.001, FP*PS: *p* = 0.895). Heart failure was more frequent in FP patients (25.5%, *p* = 0.033, VP*FP: *p* = 0.029, VP*PS: *p* = 0.740, FP*PS: *p* = 0.065). The mean serum creatinine was 1.17 ± 1.17 mg/dL, reflecting the inclusion of seven dialysis patients (4.9%). No significant differences were observed in echocardiographic indices among groups.

**Table 1:** Patients and procedural characteristics.

	All patients (*n* = 144)	FARAPULSE (*n* = 47)	PulseSelect (*n* = 48)	VARIPULSE (*n* = 49)	*p* value	
Age (years)	69.2 ± 9.9	68.1 ± 10.3	68.4 ± 10.0	71.1 ± 9.5	0.244	
Male, *n* (%)	105 (72.9)	33 (70.2)	39 (81.2)	33 (67.3)	0.268	
Paroxysmal AF, *n* (%)	98 (68.0)	37 (78.7)	23 (47.9)	38 (77.6)	0.001	VP*FP: *p* = 0.001, VP*PS: *p* < 0.001, FP*PS: *p* = 0.895
*Comorbidities*						
hypertension, *n* (%)	85 (59.0)	26 (55.3)	31 (64.6)	28 (57.1)	0.621	
Diabetes, *n* (%)	20 (13.9)	5 (10.6)	6 (12.5)	9 (18.4)	0.518	
Heart failure, *n* (%)	21 (14.6)	12 (25.5)	5 (10.4)	4 (8.2)	0.033	VP*FP: *p* = 0.029, VP*PS: *p* = 0.740, FP*PS: *p* = 0.065
History of stroke, *n* (%)	18 (12.5)	5 (10.6)	4 (8.3)	9 (18.4)	0.293	
*Medical therapy*						
Antiarrhythmic drugs, *n* (%)	78 (54.2)	31 (21.5)	25 (17.4)	22 (15.3)	0.11	
Oral Anticoagulation, *n* (%)	144 (100)	47 (100)	48 (100)	49 (100)	N.A	
*Laboratory data*						
Creatinine (mg/dL)	1.17 ± 1.17	1.1 ± 0.9	1.1 ± 1.0	1.1 ± 0.9	0.427	
eGFR (mL/min/1.73 m^2^)	60.6 ± 20.2	64.8 ± 20.9	60.1 ± 18.2	57.9 ± 21.5	0.178	
*Echocardiographic data*						
LVEF (%)	61.9 ± 12.8	59.8 ± 12.6	61.1 ± 13.0	65.3 ± 11.1	0.113	
LAD (mm)	41.4 ± 5.9	41.9 ± 4.7	41.1 ± 7.0	41.2 ± 5.5	0.633	
LAVI (mL/m^2^)	39.9 ± 14.9	37.2 ± 11.7	44.2 ± 19.1	39.2 ± 15.6	0.108	
*Procedure*						
PVI only	126 (86.3)	36 (76.6)	44 (91.7)	46 (93.9)	0.021	VP*FP: *p* < 0.001, VP*PS: *p* = 0.715, FP*PS: *p* < 0.001
Procedure time (min)	87 ± 27	91 ± 25	97 ± 26	76 ± 27	0.001	VP*FP: *p* = 0.001, VP*PS: *p* < 0.001, FP*PS: *p* = 0.895
LA dwelling time (min)	66 ± 22	71 ± 19	76 ± 21	52 ± 18	<0.001	VP*FP: *p* < 0.001, VP*PS: *p* < 0.001, FP*PS: *p* = 0.867
Application Number	34 ± 10	40 ± 6	56 ± 26	23 ± 5	<0.001	VP*FP:*p* < 0.001, VP*PS: *p* < 0.001, FP*PS: *p* = 0.689
Total fluoroscopy time (min)	22.3 ± 17.2	33.1 ± 20.9	25.8 ± 9.6	8.2 ± 6.4	< 0.001	VP*FP: *p* < 0.001, VP*PS: *p* < 0.001, FP*PS: *p* = 0.264
*Complication, n (%)*						
Cardiac tamponade, *n* (%)	1 (0.7)	0 (0)	1 (2.1)	0 (0)	0.365	
Thromboembolic events, *n* (%)	0 (0)	0 (0)	0 (0)	0 (0)	N.A	
Acute kidney injury, *n* (%)	2 (1.4)	2 (4.3)	0 (0)	0 (0)	0.123	

*Note:* The continuous variables are shown as the mean ± SD for parametric data and categorical variables as the number (%).

Abbreviations: AF, atrial fibrillation; eGFR, estimated glomerular filtration rate; LAD, left atrium diameter; LAVI, left atrial volume index; LVEF, left ventricular ejection fraction; PVI, pulmonary vein isolation.

## 7 Procedural Outcomes

Procedural characteristics of each system are also summarized in Table 1. The VP group was associated with a shorter procedure time (76 ± 27 min, *p* = 0.001) compared to the FP (91 ± 25 min) and PS (97 ± 26 min) groups (VP*FP: *p* = 0.001, VP*PS: *p* < 0.001, FP*PS: *p* = 0.895). Among the three groups, the LA dwelling time was significantly shorter in the VP group (52 ± 18 min, *p* < 0.001) than the FP (71 ± 19 min) and PS (76 ± 21 min) groups (VP*FP: *p* < 0.001, VP*PS: *p* < 0.001, FP*PS: *p* = 0.867). Additional RF ablation was significantly more frequent in the FP group, whereas the PS and VP groups were performed predominantly with PVI alone (*p* = 0.021) (VP*FP: *p* < 0.001, VP*PS: *p* = 0.715, FP*PS: *p* < 0.001). The mean number of PFA applications differed significantly: FP 40 ± 6, PS 56 ± 26, VP 23 ± 5 (*p* < 0.001) (VP*FP: *p* < 0.001, VP*PS: *p* < 0.001, FP*PS: *p* = 0.689). Total fluoroscopy time was shortest in the VP group (8.2 ± 6.4 min, *p* < 0.001) (VP*FP: *p* < 0.001, VP*PS: *p* < 0.001, FP*PS: *p* = 0.264). Complications included one cardiac tamponade in the PS group, which required surgical repair, and two cases of AKI in the FP group (in accordance with the RIFLE classification, AKI was defined as a ≥ 1.5‐fold increase in serum creatinine from the preprocedural baseline), both of which resolved with conservative management. Each of these cases was associated with stage 2−3 chronic kidney disease (CKD). No strokes or esophageal complications occurred.

## 8 Time‐Course Changes in Hemolysis and Myocardial Injury Markers

**Figure 1:**
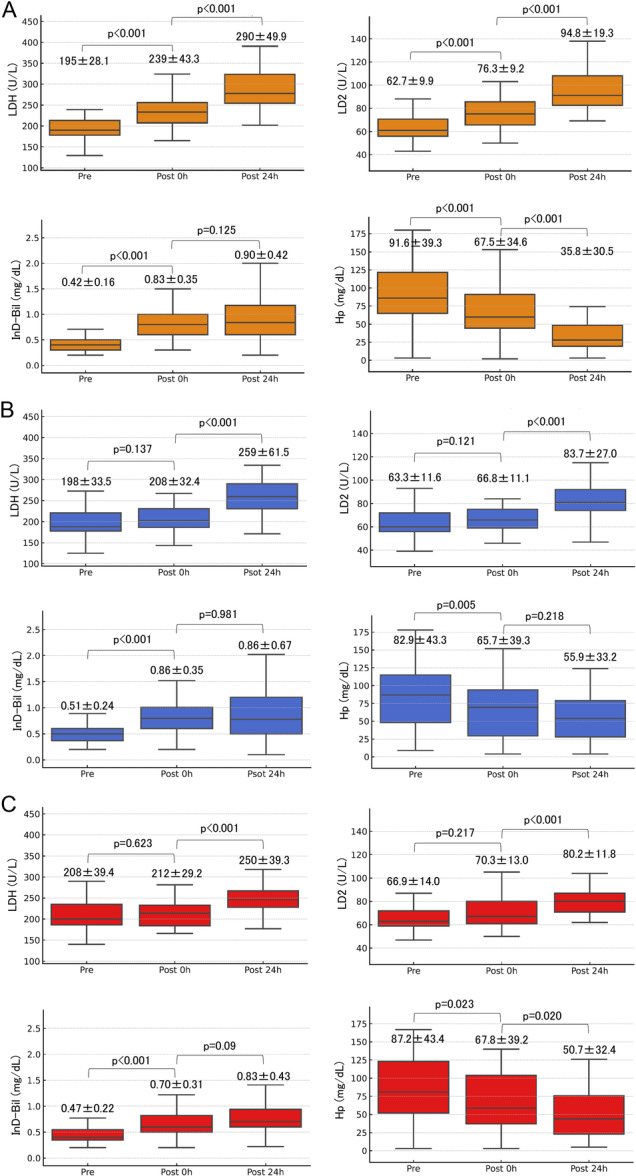
Temporal changes in hemolysis markers in the FARAPULSE group (A), PulseSelect group (B), and VARIPULSE group (C). Hemolysis markers include lactate dehydrogenase (LDH), LD isoenzyme 2 (LD2), indirect bilirubin (InD‐Bil), and haptoglobin (Hp). Values are shown at baseline, immediately post‐procedure (Post 0 h), and postoperative day 1 (Post 24 h).

As shown in Figure 1, all systems demonstrated temporal changes in hemolysis markers. FP showed a significant increase in LDH and LD2 over time, while Hp decreased significantly. In contrast, InD‐Bil showed a significant increase at Post‐0h (*p* < 0.001), but no significant change at Post‐24h (*p* = 0.125) (Figure 1A). In the PS group, hemolysis markers showed no significant change in LDH or LD2 at Post‐0h (LDH: *p* = 0.137, LD2: *p* = 0.121), but both increased significantly at Post‐24 h (*p* < 0.001). InD‐Bil and Hp showed significant changes at Post‐0 h (*p* < 0.001), but no significant changes were observed at Post‐24 h (*p* = 0.981) (Figure 1B). In the VP group, the time course change of hemolysis markers behaved similarly to the PS group. InD‐Bil is similar to the FP and PS groups.

**Figure 2:**
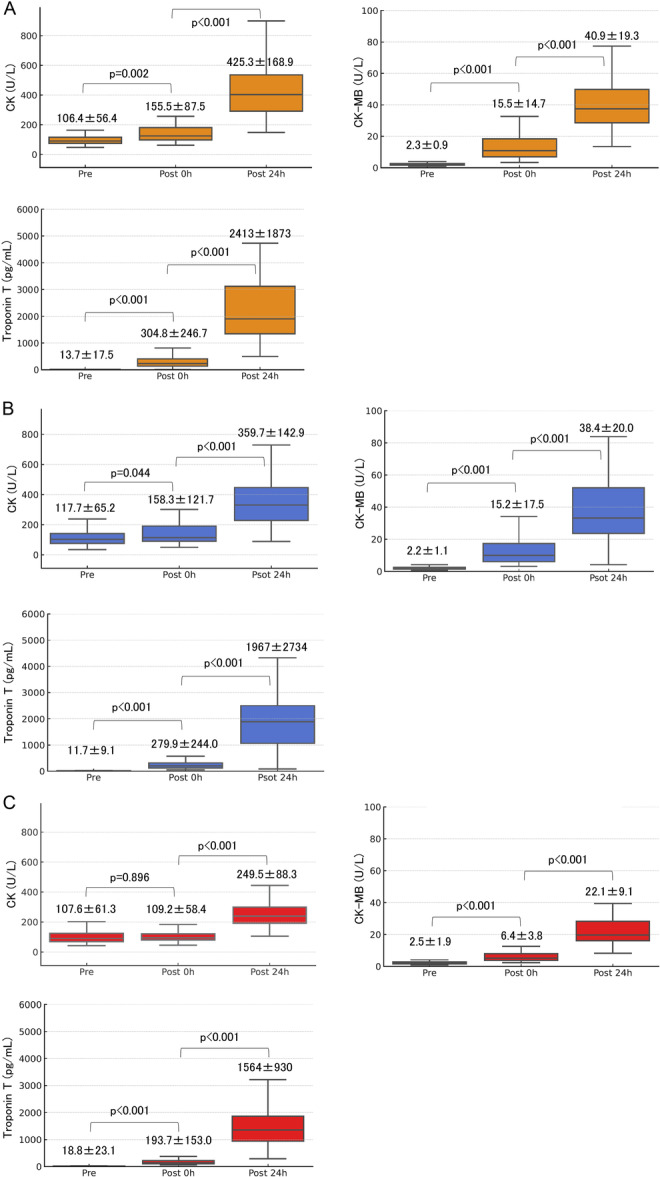
Temporal changes in myocardial injury markers in the FARAPULSE group (A), PulseSelect group (B), and VARIPULSE group (C). Myocardial injury markers include creatine kinase (CK), CK‐MB, and troponin T. Values are shown at baseline, immediately post‐procedure (Post 0 h), and postoperative Day 1 (Post 24 h).

Figure 2A‐C shows the levels of myocardial injury markers across the three systems. Myocardial injury markers increased in all groups, with VP showing the smallest early rise.

## 9 Comparison of Hemolysis and Myocardial Injury Markers Among the Systems

**Figure 3:**
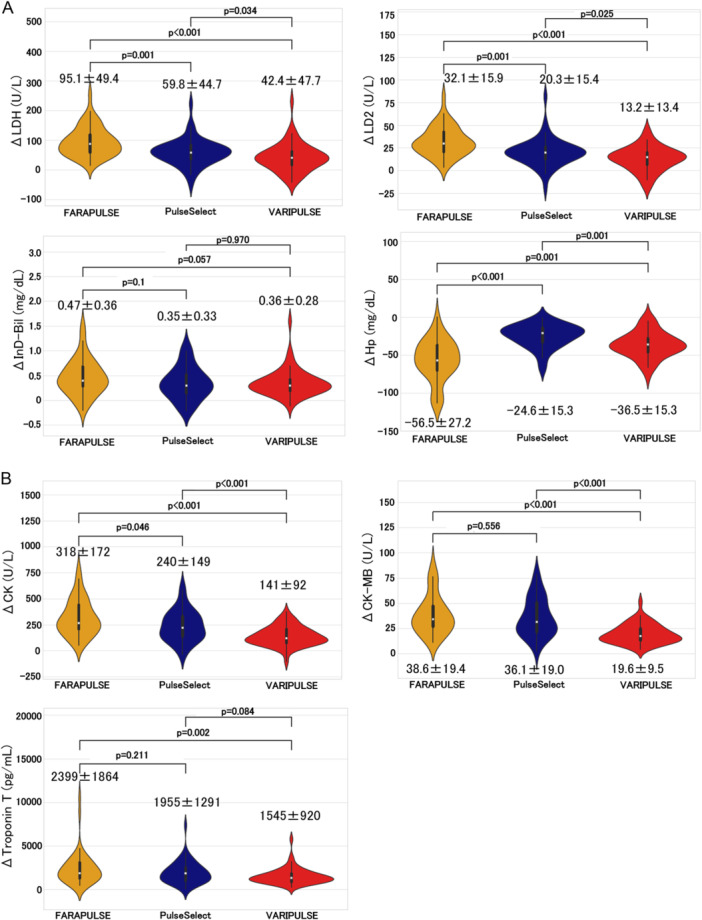
Comparison of changes in hemolysis (A) and myocardial injury markers (B) from baseline to postoperative Day 1 (Post 24 h) among the three PFA systems (FARAPULSE, PulseSelect, and VARIPULSE). Δ indicates change from baseline. Abbreviations are as in Figures 1 and 2.

As summarized in Figure 3, FP consistently demonstrated the greatest hemolysis and myocardial injury from baseline to postoperative day 1, whereas VP showed the smallest overall changes. PS exhibited intermediate results, with the least reduction in Hp but greater LDH/LD2 changes than VP. InD‐Bil did not differ significantly among groups.

## 10 Discussion

The major findings of this study can be summarized as follows:

- Procedurally, LA dwelling time and Total fluoroscopy time were shortest in the VP group. The application number was also the least in the VP group.
- With respect to complications, AKI occurred in two FP patients (4.3%), both of which improved with conservative therapy. One patient (2.1%) in the PS group experienced cardiac tamponade requiring surgical repair.
- Regarding the temporal changes in hemolysis and myocardial injury markers, most markers tended to progress from Post‐0 h to Post‐24 h, except for InD‐Bil. InD‐Bil showed no significant changes between Post‐0 h and Post‐24 h with any of the systems. Notably, an immediate postoperative increase in LDH was observed only in the FP group.
- Comparing the degree of hemolysis, FP exhibited the greatest changes, whereas PS had the smallest changes in Hp.
- For myocardial injury markers, FP and PS demonstrated similar degrees of elevation, while VP had significantly smaller increases compared to the two other systems.

## 11 Comparison of the Procedural Data Among the PFA Systems

In the PS group, a higher proportion of non‐PAF cases was included (52.1%), and additional RF ablation was more frequently performed in the FP group; both factors could have influenced the procedure time. However, the LA dwelling time, excluding the influence of additional RF applications, was shortest in the VP group. Furthermore, the VP group demonstrated the shortest total fluoroscopy time, along with the lowest number of PFA applications. The VP system is integrated with the CARTO mapping system, and its protocol required the lowest number of PFA applications. Those factors contributed to the shortest procedure and total fluoroscopy times. A known limitation of PFA procedures is the stimulation of the phrenic nerve and bronchus, which causes varying degrees of dry cough [25] leading to prolongation of the procedure time. In the present study, no muscle relaxants were administered; however, PFA was performed under deep sedation with the BIS controlled between 40 and 60. This stable deep level of sedation was considered to have contributed to a reliable PFA delivery.

## 12 Temporal Changes in Hemolysis

PFA‐induced hemolysis occurs due to physical disruption of red blood cells from steep high‐voltage pulses delivered over microsecond durations. This rapid electric field disrupts the lipid bilayer of the erythrocyte membrane, leading to irreversible electroporation [9]. PFA is considered to act through electroporation‐induced programmed cell death, namely apoptosis; however, in the present study, cell injury markers increased immediately after the procedure, suggesting that PFA may not only involve the induction of apoptosis. Kotnik et al. reported that, in addition to electroporation, PFA‐induced cell injury may also involve electropermeabilization of the cell membrane caused by the electric field, leading to cytoplasmic molecule leakage. It is suggested that this could be one of the reasons for the immediate elevation of cell injury markers observed after PFA [26].

In the present study, we measured LDH, LD2, Hp, and InD‐Bil as hemolysis markers, and CK, CK‐MB, and troponin T as myocardial injury markers. LDH, LD2, and myocardial injury markers continued to increase from immediately from Post‐0 h to Post‐24 h across all systems. In contrast, InD‐Bil exhibited a significant increase at Post‐0 h but no significant change at Post‐24 h, suggesting that direct hemolysis induced by PFA predominantly occurs during the procedure. On the other hand, since Hp continued to decrease up to Post‐24 h, this indicates that although direct hemolysis caused by PFA takes place primarily during the procedure, its effects persist until Post‐24h with all systems. Furthermore, given that the half‐life of InD‐Bil is only several hours and shorter than that of LDH, LD2, and Hp, it is reasonable to assume that its peak occurs earlier.

## 13 Differences in Hemolysis and Myocardial Injury Markers Among the PFA Systems

Overall, FP produced the largest changes in both hemolysis and myocardial injury. For PS and VP, LDH and LD2 changes were greater with PS; however, Hp—a more specific hemolysis marker—showed the smallest reduction with PS. This discrepancy suggests that LDH and LD2 changes may partially reflect myocardial injury rather than pure hemolysis. In contrast, InD‐Bil did not differ significantly among the groups, possibly due to its narrow range and susceptibility to interindividual variations in hepatic metabolism, as reported by Xuan et al. [21]. For myocardial injury, VP consistently exhibited the smallest marker changes. The reasons for these inter‐system differences are likely multifactorial. Previous studies have identified application number and tissue contact quality as key determinants of hemolysis. Poor tissue contact increases the electrode surface exposed to circulating blood, enhancing hemolysis [22, 27]. Thus, visualization of tissue contact is important in PFA. In addition, the application number is a known contributor, with a dose‐dependent relationship reported for hemolysis marker elevation [28]. Application numbers in our study were highest for PS and lowest for VP. In terms of tissue contact, FP can be shaped into basket and flower configurations, with the latter allowing direct visualization of PV compression under fluoroscopy. VP incorporates Tissue Proximity Indication (TPI) on the 3D map, enabling real‐time visualization of contact. In contrast, PS lacks a built‐in contact visualization method, relying on intracardiac echocardiography. Based on these parameters, PS—with more applications and less direct contact visualization—might have been expected to produce greater hemolysis; however, in our data, FP exhibited the most hemolysis. That suggested that the number of electrodes (FP: 20, PS: 9, VP: 10) may outweigh other factors, as more electrodes can increase the likelihood of suboptimal tissue contact for some electrodes, resulting in both increased hemolysis and myocardial injury. The lower myocardial injury observed with VP may be explained by its smaller number of electrodes, intermediate pulse output, and small number of PFA applications. With regard to differences in pulse output, FP delivers the highest voltage (1800–2000 V) among the three systems, whereas PS uses the lowest (1400–1500 V) and VP uses an intermediate voltage. Krassowska et al. performed a mathematical modeling study of electroporation in a single spherical cell, in which they reported that the number and size of pores do not significantly increase even when the pulse intensity is raised [29]. This finding may suggest that the differences in pulse output among the various PFA systems were not directly related to the extent of hemolysis or the elevation of myocardial injury markers.

## 14 Safety Considerations

In the present cohort, AKI occurred exclusively in the FP group and only in patients with pre‐existing stage 2–3 CKD. These patients improved with conservative treatment, but the findings highlight the need for careful preprocedural renal assessment when planning PFA. The single case of cardiac tamponade occurred in the PS group and was associated with a left atrial diverticulum detected on preprocedural CT, suggesting an anatomical vulnerability to perforation.

## 15 Considerations Regarding Arrhythmia Recurrence

In an exploratory analysis, arrhythmia recurrence occurred in 14.9% of patients in the FARAPULSE group, 22.9% in the PulseSelect group, and 24.5% in the VARIPULSE group, with no statistically significant difference among the three systems (*p* = 0.47). However, the present study was not powered to detect differences in clinical efficacy, and the sample size may have been insufficient to identify modest differences in recurrence rates. In addition, this was a single‐center, non‐randomized study, and potential confounders such as AF type, atrial substrate, and adjunctive ablation strategies may have influenced the results. Furthermore, follow‐up duration ranged from approximately 6 months to 1 year, and arrhythmia recurrence was evaluated using conventional follow‐up methods including Holter electrocardiography. Therefore, asymptomatic or intermittent AF episodes may have been underestimated. Recurrence after PFA may depend not only on the energy delivery system itself but also on procedural factors such as catheter contact, the number of applications, and operator experience. Because procedural dose may influence both hemolysis and lesion durability, optimizing PFA delivery to balance safety and efficacy will be an important consideration. Larger randomized multicenter studies with standardized ablation protocols and longer follow‐up are required to determine whether differences in hemolysis profiles among PFA systems translate into clinically meaningful differences in arrhythmia recurrence.

## 16 Study Limitations

This study had several limitations. First, it was conducted at a single center with a relatively small sample size, which may limit the generalizability of the findings. Second, the patient background characteristics and procedural strategies were not completely uniform across groups. In particular, the proportion of persistent AF was significantly higher in the PS group, and the frequency of concomitant linear ablation differed among systems. These differences may have influenced biomarker changes and procedural outcomes, although the primary endpoint focused on perioperative hemolysis and myocardial injury. Second, some patients in this study underwent adjunctive CTI ablation using a conventional RF catheter. To address the potential confounding influence of RF energy on hemolysis and myocardial injury, we performed a sub‐analysis limited to patients who underwent PVI alone. In this PVI‐only cohort (Supporting Information S1: Table and Figure), VARIPULSE maintained significantly shorter procedural times, and the overall trends regarding biomarker changes were unchanged; FARAPULSE continued to show the greatest degree of hemolysis, while VARIPULSE demonstrated the smallest myocardial injury. These findings suggest that the impact of RF energy used for CTI ablation did not appear to significantly influence hemolysis or myocardial injury in our cohort. Thirdly, although we excluded cases with blood sampling–related hemolysis, we cannot fully rule out minor procedural or sampling influences on biomarker levels. Similarly, LDH and LD2 are not entirely specific for hemolysis and may be elevated due to myocardial injury; we attempted to address this by including Hp and InD‐Bil, but confounding effects remain possible. Finally, device settings such as pulse output and application number were based on standard manufacturer recommendations with operator discretion for “bonus” applications. The absence of strict protocolization in these parameters may have introduced variability.

## 17 Conclusion

In this prospective comparison of three PFA systems for AF ablation, VP demonstrated the shortest LA dwelling time and total fluoroscopy time. FP was associated with the greatest hemolysis and myocardial injury, PS with the smallest reduction in Hp, and VP with the least myocardial injury. The observed differences may be influenced by various factors such as application number and tissue contact visualization capabilities. Preprocedural assessment of patient‐specific risks, particularly renal function, is essential to minimize hemolysis‐related complications such as AKI.

## Funding

The authors have nothing to report.

## Disclosure

H.M. received lecture fees from Biosense Webster Japan and Boston Scientific Japan. Our department received grant support from Boston Scientific Japan and Abbott Medical Japan.

## Ethics Statement

The study protocol was approved by the hospital's institutional review board (IRB number: 2024‐131).

## Supporting information


Supporting File 1

Supporting File 2

## Data Availability

The authors have nothing to report.
