# Supplementary material for: Comparison of HEMOlysis Markers Among Three Pulsed Field Ablation Systems: HEMO‐PFA Study
Source: J Cardiovasc Electrophysiol. 2026 Apr 11;37(6):1249–58. doi: 10.1111/jce.70343 (PMC13269869; doi:10.1111/jce.70343)

FARAPULSE
PVI-only group

Hemolysis markers

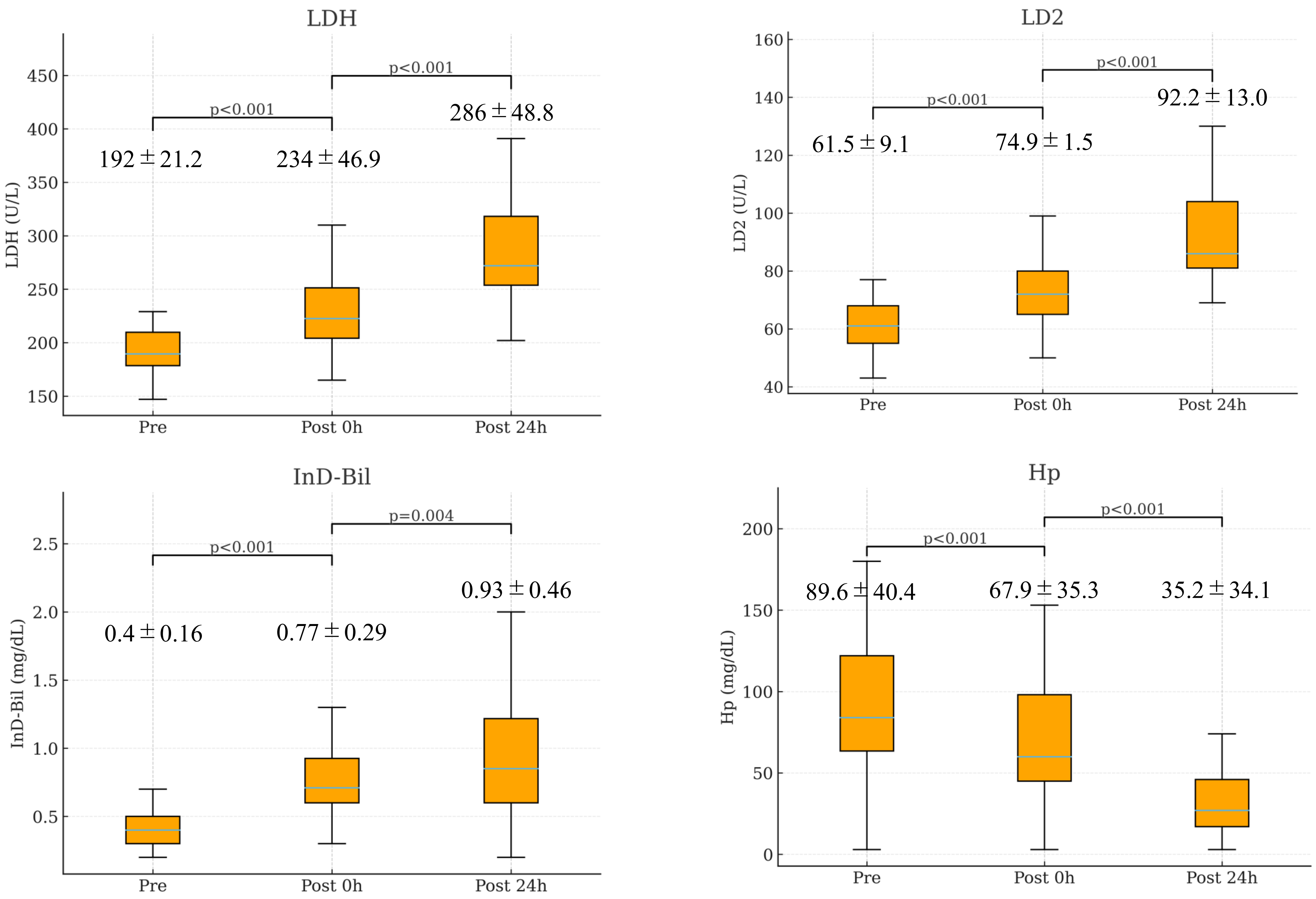

PulseSelect
PVI-only group

Hemolysis markers

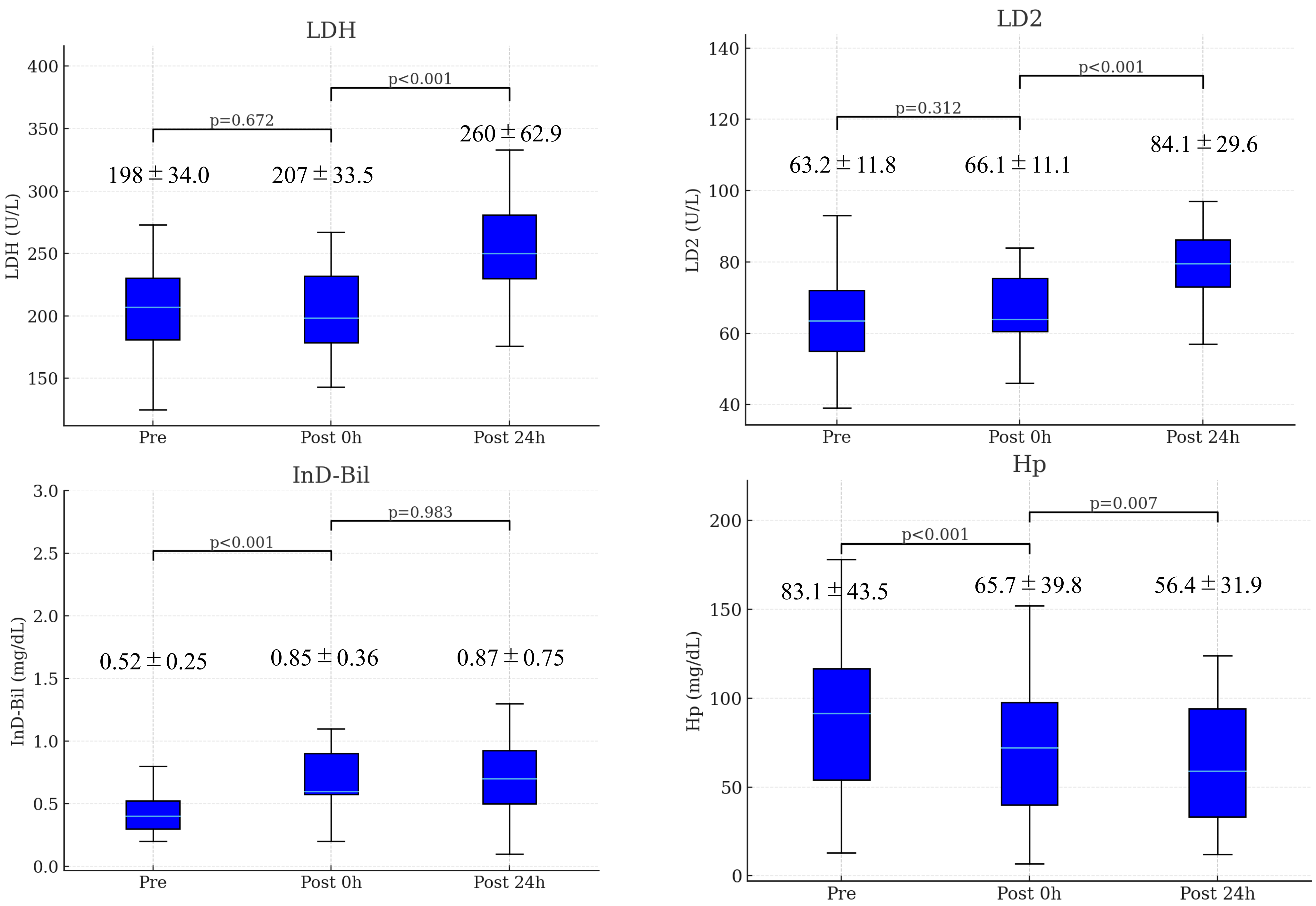

VARIPULSE
PVI-only group

Hemolysis markers

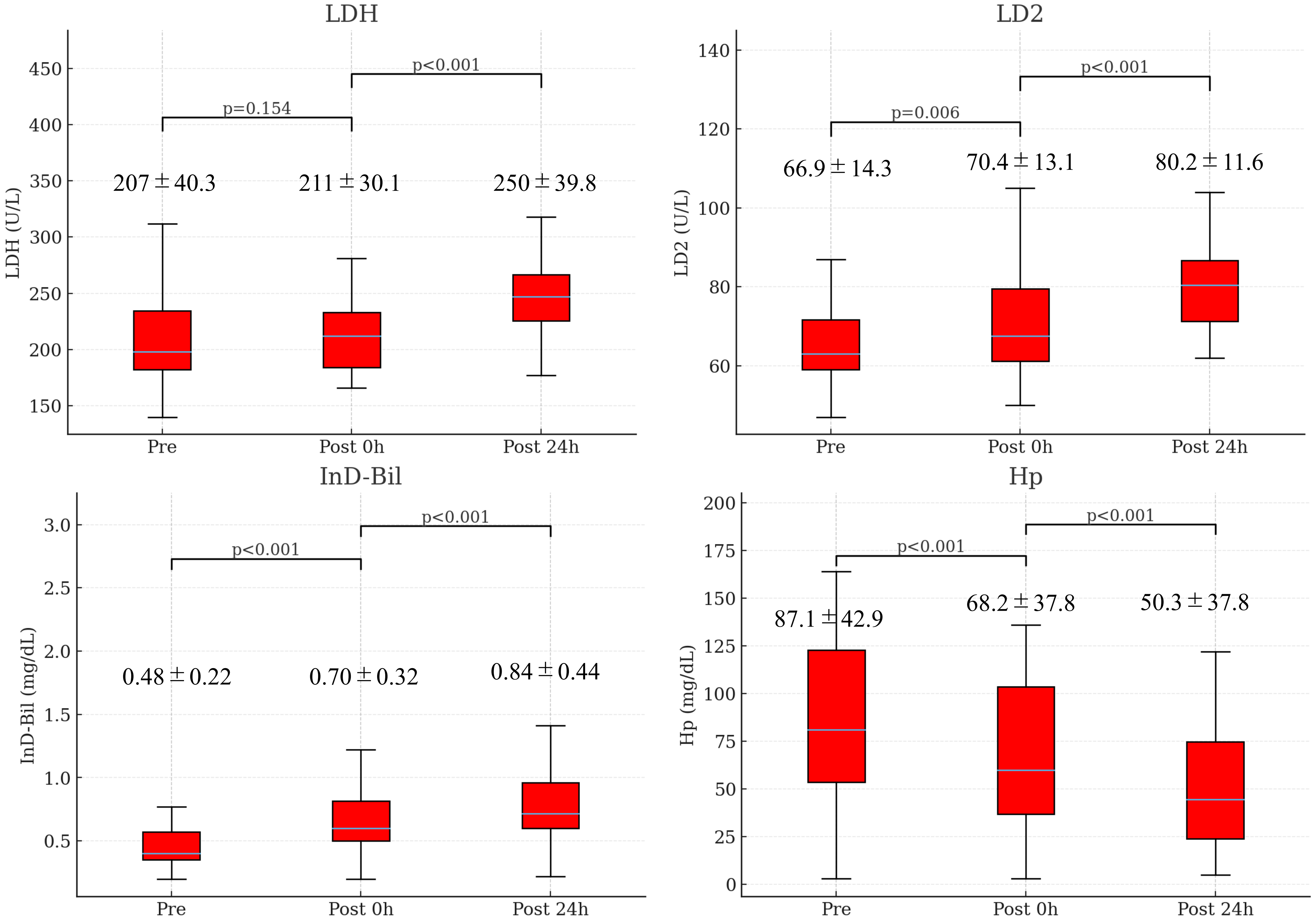

FARAPULSE
PVI-only group

Myocardial injury markers

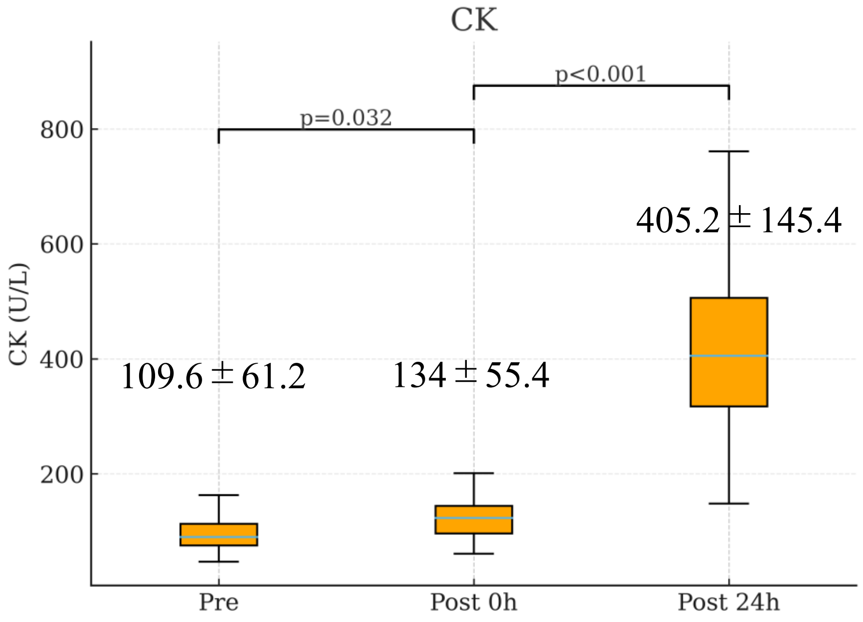

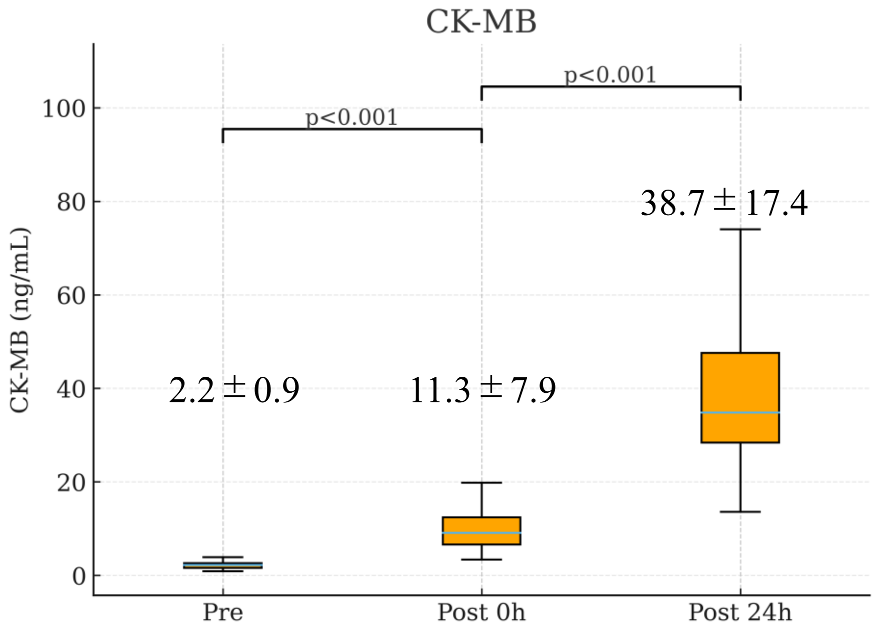

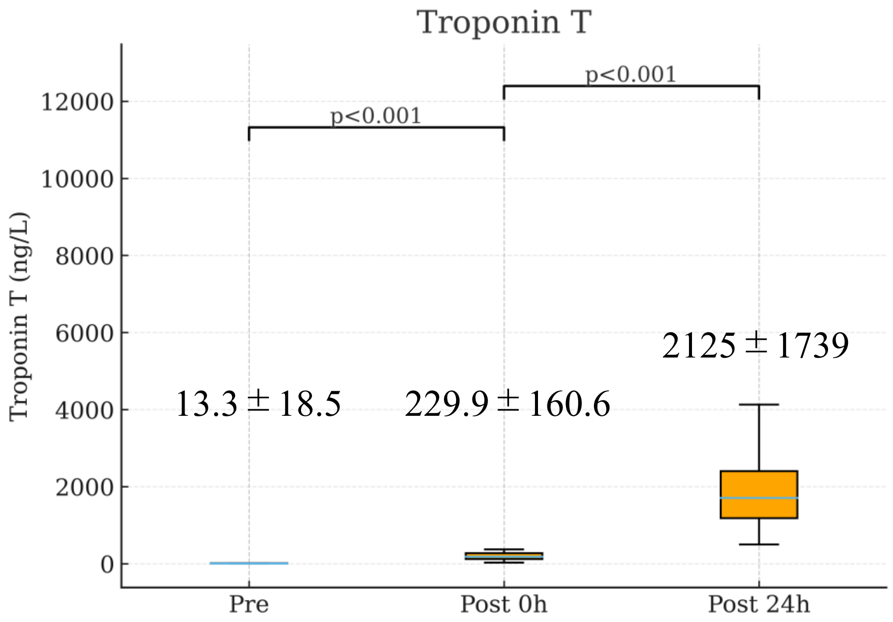

PulseSelect
PVI-only group

Myocardial injury markers

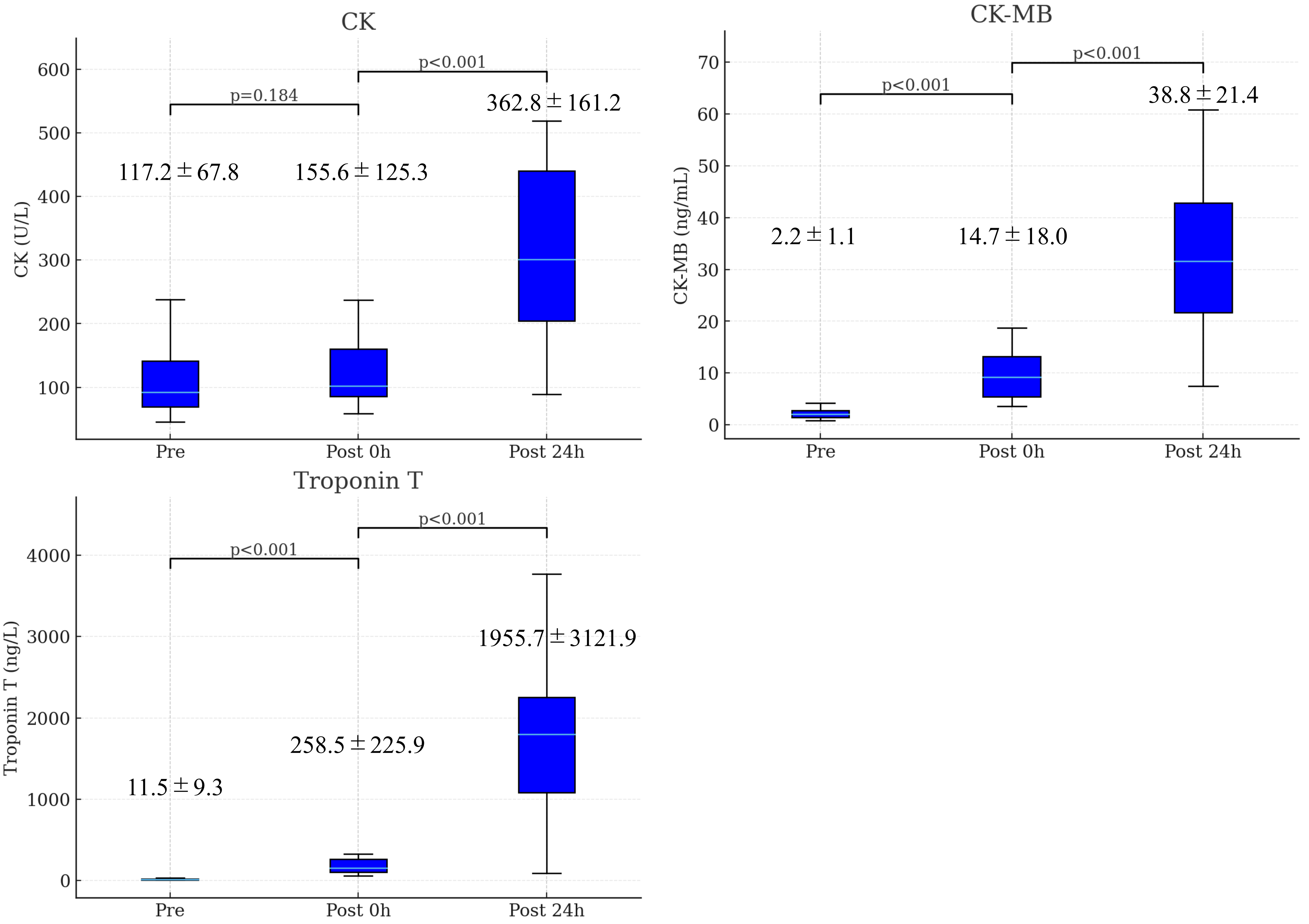

VARIPULSE
PVI-only group

Myocardial injury markers

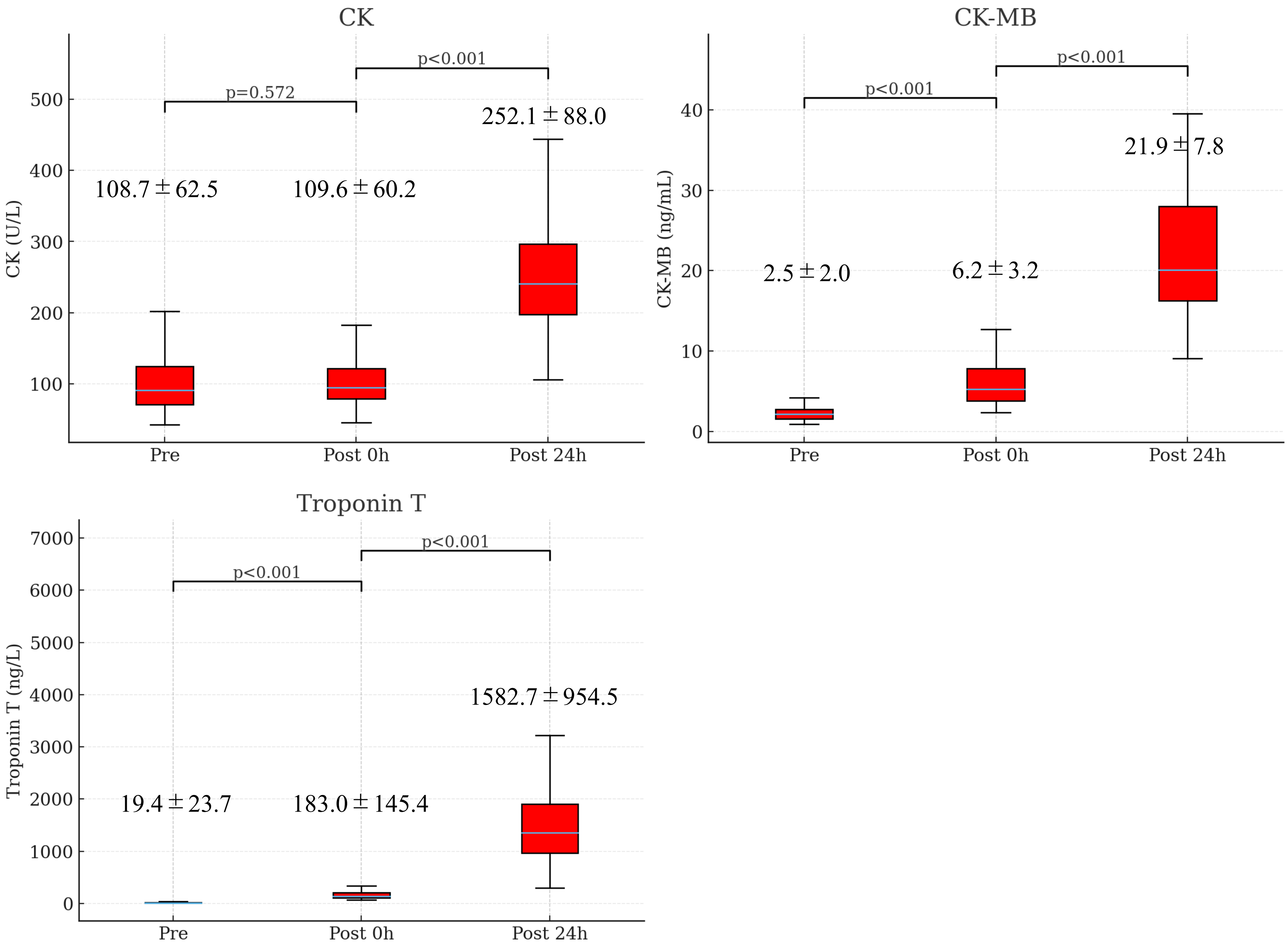

Differences in hemolysis markers among the PFA systems
(PVI-only group)

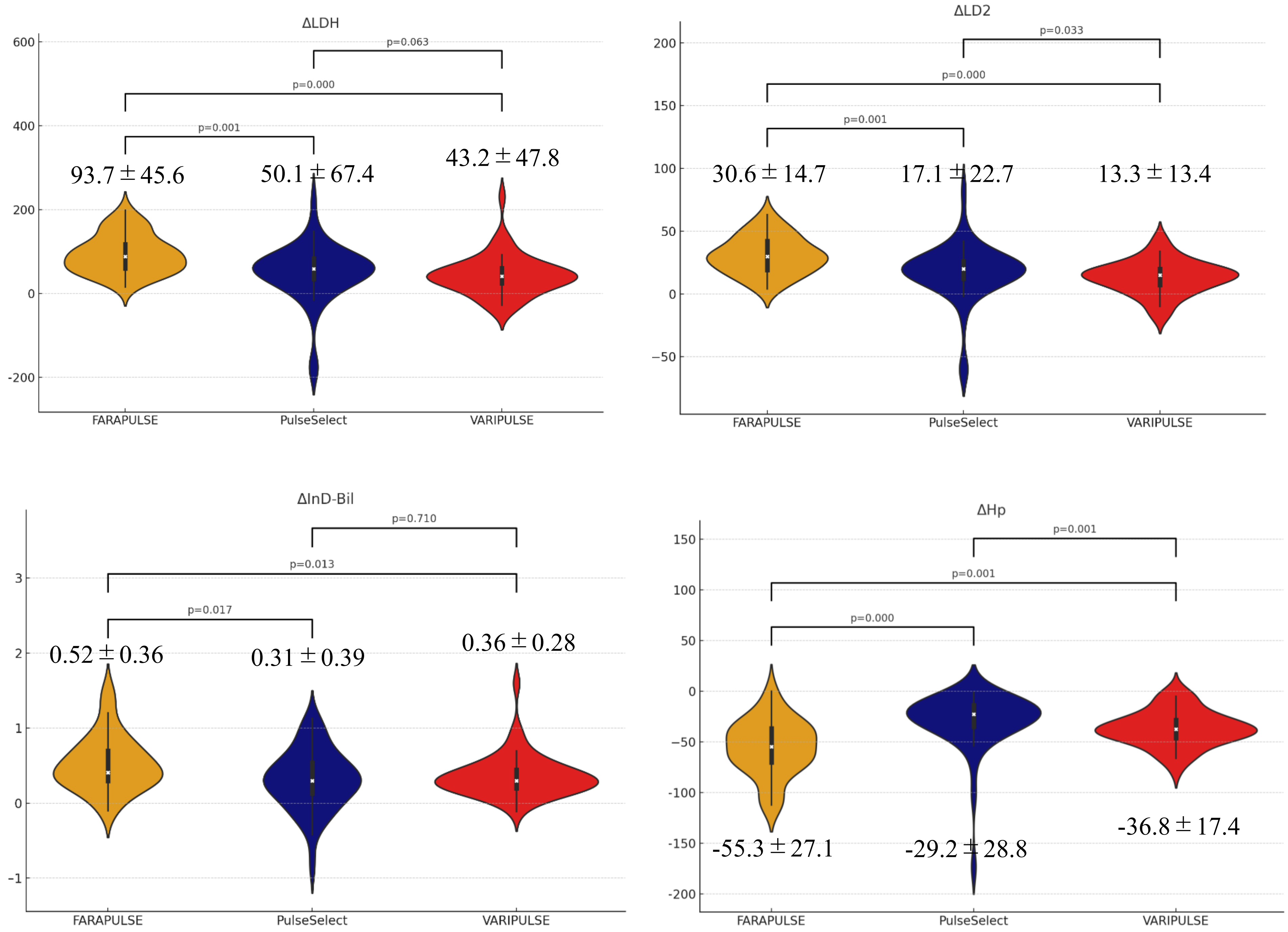

Differences in myocardial injury markers among the PFA systems
(PVI-only group)

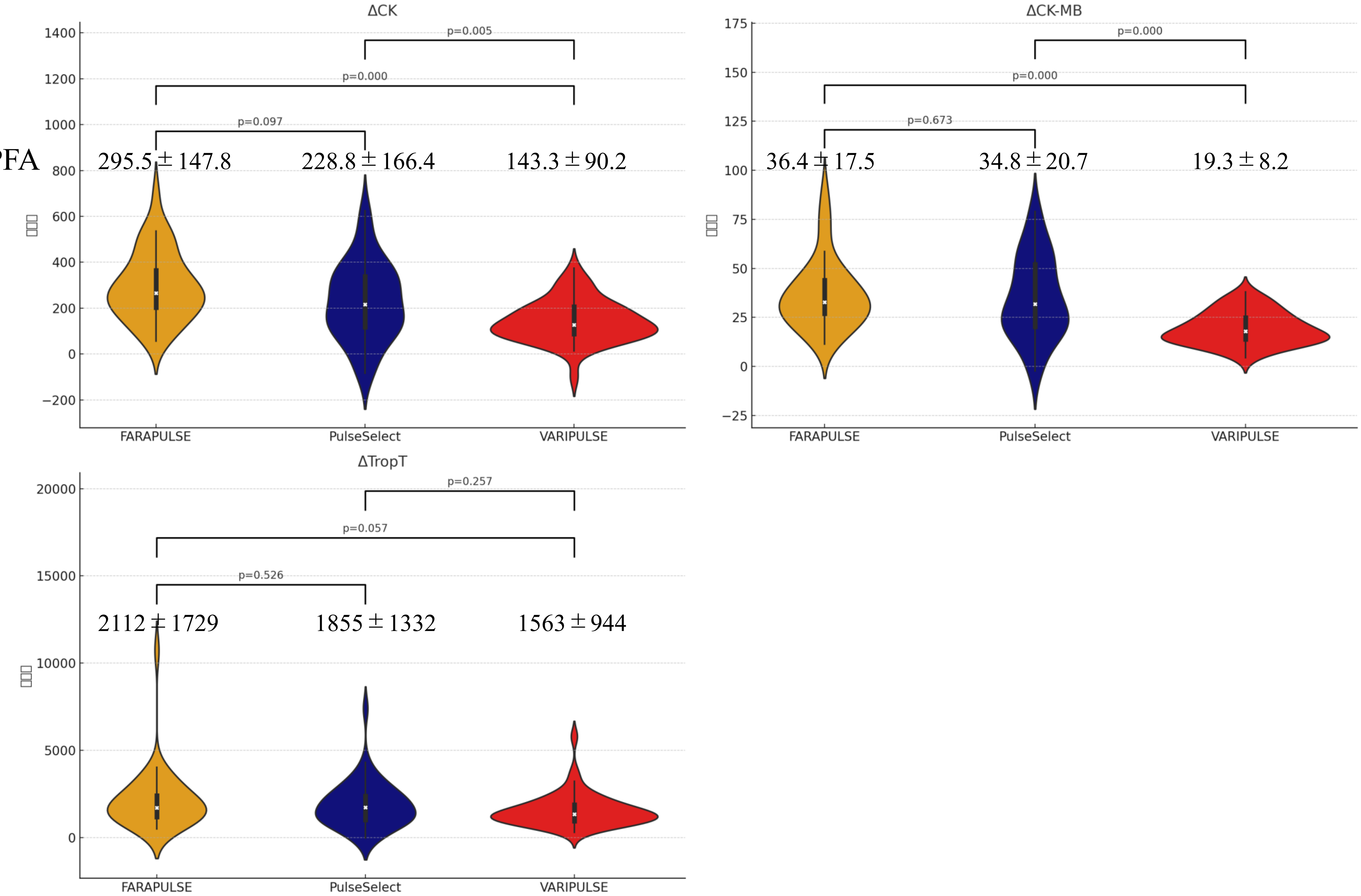

Supplement: Supplementary file 1 — Supporting File 1 [file JCE-37-1249-s001.pdf]
