# Supplementary material for: Comparison of HEMOlysis Markers Among Three Pulsed Field Ablation Systems: HEMO‐PFA Study
Source: J Cardiovasc Electrophysiol. 2026 Apr 11;37(6):1249–58. doi: 10.1111/jce.70343 (PMC13269869; doi:10.1111/jce.70343)
Supplement: Supplementary file 2 — Supporting File 2 [file JCE-37-1249-s002.docx]

|  | All patients  (n=126) | FARAPULSE  (n=36) | PulseSelect  (n=44) | VARIPULSE  (n=46) | p value |  |
| --- | --- | --- | --- | --- | --- | --- |
| Age (years) | 68.8±9.9 | 66.9±9.6 | 68.1±10.2 | 70.9±9.8 | 0.133 |  |
| Male, n (%) | 94 (74.6) | 26 (72.2) | 35 (79.5) | 33 (71.7) | 0.64 |  |
| Paroxysmal AF, n (%) | 85 (67.5) | 27 (75.0) | 21 (47.7) | 37 (80.4) | 0.002 | VP*FP:p=0.555, VP*PS:p<0.001, FP*PS:p=0.013 |
| Comobidities |  |  |  |  |  |  |
| hypertension, n (%) | 85 (59.0) | 16 (44.4) | 27 (61.3) | 27 (58.7) | 0.275 |  |
| Diabetes, n (%) | 17 (13.5) | 4 (11.1) | 5 (11.3) | 8 (17.4) | 0.623 |  |
| Heart failure, n (%) | 20 (15.8) | 11 (30.5) | 5 (11.3) | 4 (8.7) | 0.016 | VP*FP:p=0.012, VP*PS:p=0.673, FP*PS:p=0.032 |
| History of Stroke, n (%) | 16 (12.7) | 4 (11.1) | 4 (9.1) | 8 (17.4) | 0.469 |  |
| Medical therapy |  |  |  |  |  |  |
| Antiarrhythmic drugs, n (%) | 69 (54.7) | 25 (69.4) | 23 (52.3) | 21 (45.6) | 0.091 |  |
| Oral Anticoagulation, n(%) | 126 (100) | 47 (100) | 48 (100) | 49 (100) | N.A |  |
| Laboratory data |  |  |  |  |  |  |
| Creatinine (mg/dL) | 1.14±0.98 | 1.1±0.9 | 1.1±1.0 | 1.2±0.9 | 0.305 |  |
| Echocardiographic data |  |  |  |  |  |  |
| LVEF (%) | 61.8±13.1 | 58.5±14.6 | 61.6±13.2 | 64.7±11.3 | 0.161 |  |
| LAD (mm) | 41.3±5.6 | 42.3±4.3 | 40.6±6.2 | 41.2±5.6 | 0.442 |  |
| LAVI (mL/m^2^) | 38.7±13.5 | 35.1±4.2 | 42.5±13.2 | 38.5±15.6 | 0.122 |  |
| Procedure |  |  |  |  |  |  |
| Procedure time (min) | 83±24 | 86±21 | 94±23 | 74±26 | <0.001 | VP*FP:p=0.012, VP*PS:p<0.001, FP*PS:p=0.601 |
| LA dwelling time (min) | 65±21 | 71±19 | 75±20 | 51±17 | <0.001 | VP*FP:p<0.001, VP*PS:p<0.001, FP*PS:p=0.888 |
| Application Number | 34±10 | 40±6 | 39±7 | 23±5 | <0.001 | VP*FP:p<0.001, VP*PS:p<0.001, FP*PS:p=1.0 |
| Total fluoroscopy time (min) | 21.1±17.1 | 33.5±22.5 | 24.3±8.2 | 8.0±6.4 | <0.001 | VP*FP:p<0.001, VP*PS:p<0.001, FP*PS:p=1.0 |
| Complication, n (%) |  |  |  |  |  |  |
| Cardiac tamponade, n (%) | 1 (0.8) | 0 (0) | 1 (2.3) | 0 (0) | 0.391 |  |
| Thromboembolic events, n (%) | 0 (0) | 0 (0) | 0 (0) | 0 (0) | N.A |  |
| Acute kidney injury, n (%) | 1 (0.8) | 1 (2.8) | 0 (0) | 0 (0) | 0.284 |  |

Supplemental table. Patients and procedural characteristics in the PVI-only group

The continuous variables are shown as the mean ± SD for parametric data and categorical variables as the number (%).

Abbreviations: AF, atrial fibrillation; LVEF, left ventricular ejection fraction; LAD, left atrium diameter; LAVI, left atrial volume index; PVI, pulmonary vein isolation
